# PReS-FINAL-2145: MRP8/14 serum complexes as predictor of response to etanercept treatment in juvenile idiopathic arthritis

**DOI:** 10.1186/1546-0096-11-S2-P157

**Published:** 2013-12-05

**Authors:** J Anink, MH Otten, LA Van Suijlekom-Smit, MA Van Rossum, KM Dolman, EP Hoppenreijs, R Ten Cate, T Vogl, D Foell, J Roth, D Holzinger

**Affiliations:** 1 Paediatrics and Paediatric Rheumatology, Erasmus MC Sophia Children's Hospital, Rotterdam, Netherlands; 2 Paediatrics and Paediatric Rheumatology, Academic Medical Centre and Reade Institute, Rotterdam, Netherlands; 3 Paediatrics and Paediatric Rheumatology, St. Lucas Andreas Hospital and Reade Institute, Amsterdam, Netherlands; 4 Paediatrics and Paediatric Rheumatology, St. Maartenskliniek and Radboud University Medical Centre, Nijmegen, Netherlands; 5 Paediatrics and Paediatric Rheumatology, Leiden University Medical Centre, Leiden, Netherlands; 6 Institute of Immunology, University Hospital Muenster, Muenster, Germany; 7 Germany Interdisciplinary Centre for Clinical Research IZKF, University Hospital Muenster, Muenster, Germany; 8 Department of Paediatric Rheumatology and Immunology, University Children's Hospital Muenster, Muenster, Germany; 9 Germany Interdisciplinary Centre for Clinical Research IZKF, University of Muenster, Muenster, Germany

## Introduction

Biological therapy has dramatically improved the treatment of patients with JIA. However, there is still a group of patients that shows a lack of clinical response to this treatment. The use of robust predictive markers of response to identify individuals who are likely to respond to etanercept may provide guidance in optimizing treatment strategies.

## Objectives

To test the ability of MRP8/14 serum levels to differentiate between responders and non-responders to etanercept before start of treatment, and to correlate longitudinal follow-up of these markers with response to treatment.

## Methods

Samples were collected from 71 JIA patients (43 polyarthritis (12 RF positive), 18 extendend oligoarthritis, 3 persistent oligoarthritis, 1 enthesitis related arthritis, 6 psoriatic arthritis) included in the Dutch Arthritis and Biologics in Children (ABC) Register treated with etanercept. The patients were categorized into responders (acrpedi≥50) (n = 55) and non-responders (acrpedi≤50) (n = 16) according to the acrpedi response criteria and Wallace criteria of disease activity. Serum concentrations of MRP8/14 complexes were measured by ELISA at start of etanercept and in 34 patients also after start of treatment. Non-parametric tests were used to analyse the data.

## Results

Before initiation of etanercept treatment, responders showed significantly higher levels of MRP8/14 serum complexes compared to non-responders (p < 0.001). Univariate analysis showed no association between MRP8/14 and JIA disease activity (JADAS10) at baseline. In non-responders levels did not significantly change after initiation of treatment whereas levels decreased in responders (p < 0.001).

## Conclusion

High levels of baseline serum MRP8/14 have prognostic value in predicting a subgroup of JIA patients who will respond well to etanercept treatment. Decrease of MRP8/14 after initiation of treatment is associated with response to treatment.

## Disclosure of interest

J. Anink: None declared., M. Otten Grant/Research Support from: Abbott, Novartis, Roche, Pfizer, Consultant for: Roche, L. Van Suijlekom-Smit Grant/Research Support from: Dutch Board of Health Insurances, Dutch Arthritis Association, Pfizer, Abbott, Consultant for: Roche, Novartis, M. Van Rossum: None declared., K. Dolman: None declared., E. Hoppenreijs: None declared., R. Ten Cate Grant/Research Support from: Pfizer, Consultant for: Pfizer, T. Vogl: None declared., D. Foell: None declared., J. Roth: None declared., D. Holzinger Grant/Research Support from: Pfizer.

